# Do cognitive dysfunction and fatigue impact illness perception in people living with multiple sclerosis?

**DOI:** 10.1055/s-0046-1817038

**Published:** 2026-03-11

**Authors:** Raquel Portugal-Haraki, Paloma Peter Travassos Zaidan, Jessica Monique Dias Alencar, Enedina Maria Lobato de Oliveira

**Affiliations:** 1 Universidade Federal de São Paulo, Escola Paulista de Medicina, Departamento de Neurologia e Neurocirurgia, São Paulo SP, Brazil.

**Keywords:** Multiple Sclerosis, Cognitive Dysfunction, Fatigue

## Abstract

**Background:**

Multiple sclerosis (MS) is a chronic disease with an unpredictable clinical course, presenting both recurrent relapses and progressive worsening over time. People living with MS (pwMS) are impacted physically, psychologically, and socially. Illness perception refers to a person's cognitive and emotional representations of their disease, which can impact their psychophysical well-being and treatment adherence.

**Objective:**

To assess the illness perception of pwMS and determine how it is impacted by cognitive dysfunction and fatigue.

**Methods:**

In this cross-sectional pilot survey study, the Brief Illness Perception Questionnaire (B-IPQ), Montreal Cognitive Assessment (MoCA-BR), Modified Fatigue Impact Scale (MFIS-BR), and Multiple Sclerosis Quality of Life Questionnaire (MSQOL-54) were used. Demographic and clinical data, as well as the Expanded Disability Status Scale (EDSS), were collected.

**Results:**

A total of 30 RRMS patients (66.7% of female subjects, with a mean age of 36.8 ± 1.8 years) were enrolled. The mean score on the B-IPQ scale was of 30.2 ± 8.9 points, with 40% presenting a threat perception of the disease. No significant correlation was observed between cognitive performance and illness perception (r = −0.058; *p* = 0.761). However, significant correlations were found between illness perception and all fatigue domains (total: r = 0.578; *p* = 0.001; physical: r = 0.594; *p* = 0.001; psychosocial: r = 0.672; *p* < 0.001), except the cognitive domain (r = 0.360; *p* = 0.051). The EDSS and MSQOL-54 scores also demonstrate significant correlations with illness perception (EDSS = 2.30; *p* = 0.034; MSQOL-54 physical: r = −0.776; *p* < 0.001; MSQOL-54 mental: r = −0.704; *p* < 0.001).

**Conclusion:**

Illness perception in MS is influenced by patients' fatigue, quality of life, and disability levels, but it does not appear to be affected by cognition.

## INTRODUCTION

Multiple sclerosis (MS) is an autoimmune, inflammatory, and neurodegenerative disease that affects more than 2 million people worldwide, being the leading cause of neurological disability in young adults. 1 It typically presents as recurrent episodes of inflammation in the central nervous system in various locations. However, it can also present with progressive worsening over time. The symptoms are diverse and may affect various neurological functions, including mobility, motor function, vision, sexual function, and cognition. As a a chronic disease with an unpredictable clinical course that requires long-term treatment, 2 MS impacts the lives of patients in multiple ways, including physical, psychological, and social aspects. Consequently, people with MS (pwMS) tend to have a poorer quality of life. 2 3

Cognitive dysfunction is common in multiple sclerosis and contributes to the disability caused by the disease. It is estimated that 40 to 60% of pwMS experience some cognitive impairment. It usually manifests as a mild to moderate impairment, with processing speed and memory being the most affected domains. 4 5 Being related to psychological mechanisms, changes in cognition may influence an individual's perception of their disease, either by underestimating or overestimating the perceived threat. 6 7

Fatigue is a highly disabling symptom, reported by up to 80% of individuals with MS. It is defined as a subjective lack of physical and/or mental energy that interferes with daily activities. Due to its inherently subjective nature, Bassi et al. have explored whether psychological factors, such as illness perception, could contribute to its development. They demonstrated a correlation between certain illness perception factors and fatigue. 8

Illness perception may be defined as patients' cognitive and emotional representations of their disease. 3 According to Leventhal's self-regulation model, individuals with illnesses construct personal representations of their condition based on several aspects: the label assigned to the disease (identity), assumptions about its course (timeline), determinants (causes), beliefs about its impact on daily life (consequences), controllability (control), understanding of the disease (coherence), and the emotional effect of the illness (emotional representations).

The response to each of these factors can influence the coping strategies adopted and patients' emotional response to their condition. 1 3 How patients perceive their illness also affects their psychophysical well-being, quality of life, and treatment adherence. Research involving pwMS has shown a correlation between negative illness perceptions and lower quality of life, high levels of depression and anxiety, greater functional impairment, more negative beliefs about treatment efficacy, and lower treatment adherence. 1 3 9

The present study aimed to assess the illness perception of pwMS and determine the impact of cognitive impairment. Additionally, we examined the effects of fatigue and how illness perception affects quality of life.

## METHODS

### Patient selection

This is a cross-sectional pilot survey study conducted with patients diagnosed with relapsing-remitting MS (RRMS) according to the 2017 McDonald criteria, aged 18 years or older, and under regular follow-up for at least six months at the Neuroimmunology Clinic of Hospital São Paulo, Escola Paulista de Medicina, Universidade Federal de São Paulo (UNIFESP). Patients were screened and further recruited during the regular follow-up appointments.

We excluded patients with less than 8 years of schooling and those diagnosed with dementia according to the National Institute on Aging and Alzheimer's Association (NIA-AA) and the Brazilian Academy of Neurology (Academia Brasileira de Neurologia, ABN, in Portuguese) criteria, or those who had a Montreal Cognitive Assessment (MoCA) score below the cutoff for dementia based on the level of schooling, as suggested by the ABN. 10 11 Since pregnancy may influence cognitive functions, particularly executive functioning, 12 we excluded pregnant participants from the study.

Additionally, we also excluded patients with psychiatric conditions, regardless of treatment, or other relevant clinical comorbidities, such as uncontrolled diabetes, hypothyroidism, chronic obstructive pulmonary disease (COPD), severe cardiac insufficiency, chronic kidney disease, a relapse or systemic corticosteroid treatment (oral or intravenous) within 3 months, and those who refused to participate.

As this is a pilot study, a sample size of 30 patients was deemed sufficient, given that the sampling distribution of the mean approaches normality for samples of this magnitude.

Participants were assessed using validated scales in Brazilian Portuguese to evaluate illness perception, cognition, fatigue, and quality of life. The assessments were conducted in person and supervised by the authors, taking 2 hours to complete, on average. All participants provided a signed informed consent form. The study was approved by the Research Ethics Committee of UNIFESP, under CAAE number 72803323.4.0000.5505.

### Questionnaires and scales

We used the Brief Illness Perception Questionnaire (B-IPQ), a summarized version of the IPQ-R comprising nine items, to assess illness perception. This self-administered questionnaire contains seven Likert scale questions, with scores ranging from 0 to 10 points. The final score ranges from 0 to 70 points, and a result above 33 indicates a relevant threat perception. 13

The Montreal Cognitive Assessment (MoCA-BR) was chosen to evaluate cognitive impairment. It consists of 12 subitems designed to assess different cognitive domains (memory, orientation, attention, executive function, language, and visuoconstructive ability), with a maximum score of 30 points. 14

We used the Brazilian version of the Modified Fatigue Impact Scale (MFIS-BR) to assess fatigue. This self-administered scale comprises 21 Likert-format questions, divided into three domains: physical (9 items), cognitive (10 items), and psychosocial (2 items). The total score is calculated by summing the scores across the three domains and ranges from 0 to 84 points. A score above 38 points indicates fatigue, with higher results indicating greater fatigue. 15

The Brazilian version of the Multiple Sclerosis Quality of Life Questionnaire (MSQOL-54) was used to evaluate quality of life. This self-administered questionnaire consists of 54 Likert-scale questions, divided into two domains (Physical Health and Mental Health), ranging from 0 to 100 points, with higher scores indicating better quality of life. 16 Additionally, we collected demographic and clinical data, including disease duration and disability status, assessed by the Expanded Disability Status Scale (EDSS). 17

### Statistical analysis

We performed a descriptive analysis to characterize the study population, including sex, age, level of schooling, disease duration, EDSS, and the questionnaire scores, using summary measures or frequency distributions. The Kolmogorov-Smirnov test was used to assess data normality, and Student's t-test was employed to compare MoCA-BR's mean scores by illness perception status.

To examine the correlation between illness perception, cognitive ability, fatigue, and quality of life, we performed Pearson's correlation analyses using the corresponding scores: B-IPQ for illness perception, MoCA-BR for cognitive ability, MFIS-BR for fatigue, and MSQOL-54 for quality of life. The effects of disease duration and EDSS on illness perception were evaluated using multivariate linear regression for the outcome of continuous B-IPQ score and multivariate logistic regression for the outcome of the presence of relevant threat perception (B-IPQ > 33).

A 5% significance level ( *p* -value < 0.05) was adopted for all statistical tests. The analyses were conducted using the IBM SPSS Statistics for Windows (IBM Corp.), version 20.0.

## RESULTS

**Figure 1:**
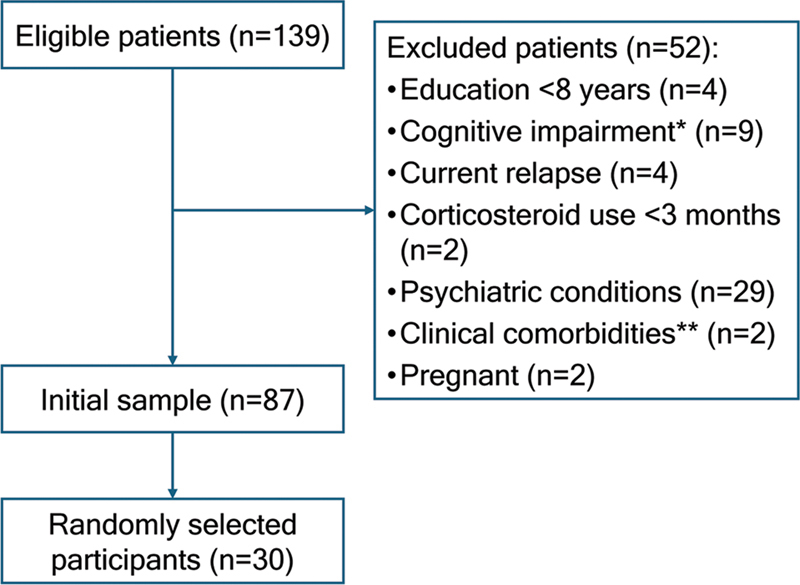
Notes: *Diagnosis of dementia according to the diagnostic criteria of the National Institute on Aging and Alzheimer's Associ8ation (NIA-AA) and the Brazilian Academy of Neurology (ABN), or those who had a Montreal Cognitive Assessment (MoCA) score below the cutoff for dementia based on the level of schooling suggested by ABN; **Patients who had other relevant clinical comorbidities. Recruitment of participants.

A total of 139 individuals were eligible for the study. Due to the exclusion criteria, 87 patients remained in the initial pool with the same probability of being selected. Finally, 30 were chosen through simple random sampling to complete the questionnaires and research scales during their regular appointments, as shown in Figure 1 .

The studied population had a mean age of 36.8 ± 11.8 years), with 66.7% of the participants being women, and a mean of 12.9 ± 1.8 years) of schooling. The mean disease duration was of 11.6 ± 7.6 years), and the median EDSS score was 2.0 points (interquartile range [IQR] = 1.4–2.6 points), as shown in Table 1 .

**Table 1:** Patients' demographics and clinical characteristics

Demographic and clinical data (n = 30)	Results
**Sociodemographic characteristics**	
**Female sex: n (%)**	20 (66.7%)
**Mean age**	36.8 ± 11.8
**Mean years of schooling**	12.9 ± 1.8
**Clinical characteristics**	
**MS phenotype: n (%)**	
RRMS	30 (100%)
**Mean disease duration (years)**	11.6 ± 7.6
**Mean time since last relapse (years)**	5.8 ± 4.5
**Mean number of relapses**	4.4 ± 3.5
**Disease-modifying drug: n (%)**	
Interferon	2 (6.7%)
Copaxone	1 (3.3%)
Teriflunomide	2 (6.6%)
Dimethyl fumarate	6 (20%)
Fingolimod	8 (26.7%)
Natalizumab	3 (10%)
Alentuzumab	2 (6.7%)
Ocrelizumab	5 (16.7%)
None	1 (3.3%)
**EDSS score: median (IQR)**	2.0 (1.4–2.6)
Pyramidal	0.5 (0–1)
Cerebellar	0 (0–0)
Brainstem	0 (0–0)
Sensitive	0 (0–1)
Sphincter	0 (0–0.75)
Visual	1 (0–1)
Mental	0 (0–0)
**Neuropsychological tests**	
**Mean B-IPQ score**	30.2 ± 8.9
Relevant threat perception (B-IPQ > 33): n (%)	12 (40%)
**Mean MoCA-BR score**	25.7 ± 2.1
**Mean MFIS-BR score**	
Total	35.8 ± 18.0
Cognitive	16.8 ± 8.5
Physical	15.7 ± 9.8
Psychosocial	3.3 ± 2.2
**Mean MSQOL-54 score**	
Physical Health	66.8 ± 18.4
Mental Health	63.8 ± 20.7

Abbreviations: B-IPQ, Brief Illness Perception Questionnaire; EDSS, Expanded Disability Status Scale; IQR, interquartile range (percentile 25–75); MFIS-BR, Brazilian version of the Modified Fatigue Impact Scale; MoCA-BR, Montreal Cognitive Assessment; MS, multiple sclerosis; MSQOL-54, Multiple Sclerosis Quality of Life Questionnaire; RRMS, relapsing-remitting multiple sclerosis.

Regarding illness perception, the mean score on the B-IPQ scale was of 30.2 ± 8.9 points, with 40.0% of our sample presenting a threat perception regarding the disease, as shown in Table 1 . The performance of the studied population on the cognitive (MoCA-BR), fatigue (MFIS), and quality of life (MSQOL-54) scales is presented in Table 1 .

**Figure 2:**
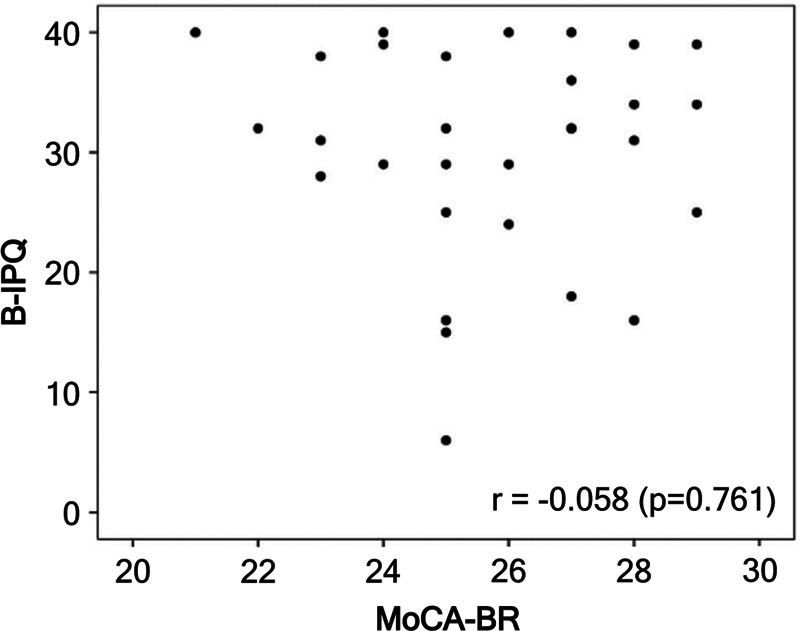
Abbreviations: B-IPQ, Brief Illness Perception Questionnaire; MoCA-BR, Montreal Cognitive Assessment. Note: A linear association via Pearson's correlation coefficient was used. A 5% significance level ( *p* < 0.05) was adopted. Scatter plot of MoCA-BR and B-IPQ.

Correlation analysis between cognitive performance, measured by MoCA-BR, and illness perception, assessed using the B-IPQ scale, showed no significant correlation (r = −0.058; *p* = 0.761), as shown in Figure 2 . Furthermore, no significant differences were found in the MoCA-BR mean scores based on threat perception status (relevant threat perception: no – 25.6 ± 1.9 versus yes – 25.9 ± 2.5; *p* = 0.658).

**Figure 3:**
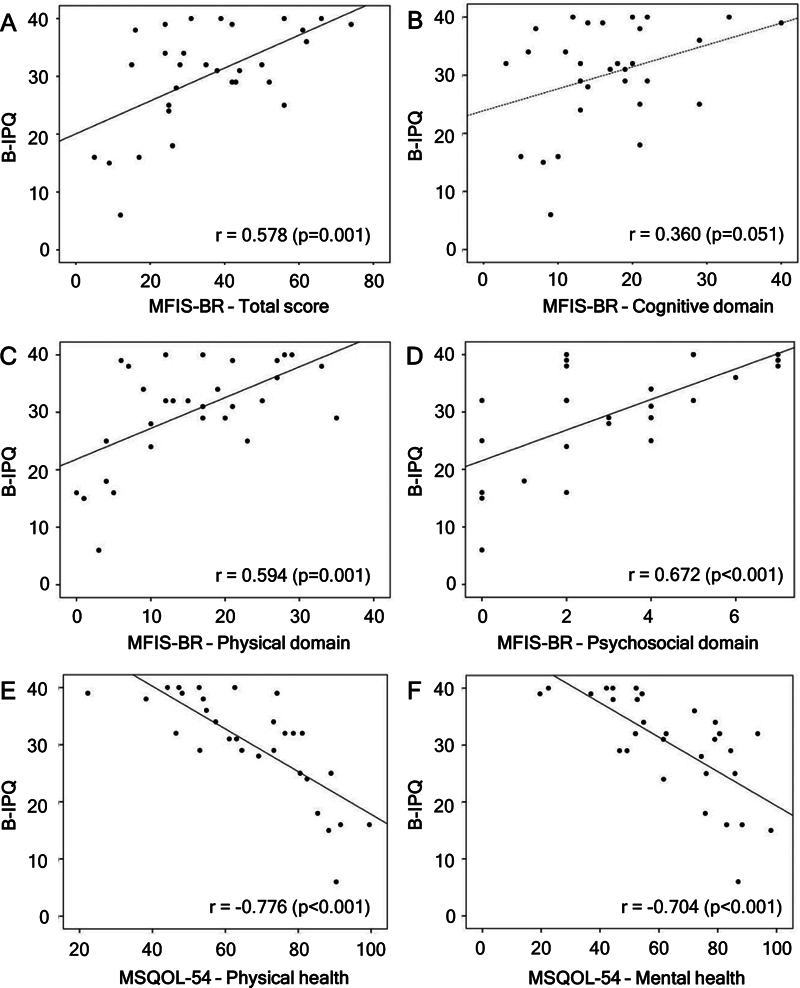
Abbreviations: B-IPQ, Brief Illness Perception Questionnaire; MFIS-BR, Brazilian version of the Modified Fatigue Impact Scale; MSQOL-54, Multiple Sclerosis Quality of Life Questionnaire. Note: A linear association via Pearson's correlation coefficient was used. A 5% significance level ( *p* < 0.05) was adopted. Scatter plot of MFIS-BR, MSQOL-54 and B-IPQ.

A significant correlation was observed between illness perception (B-IPQ) and fatigue (MFIS-BR total score and domains). There was a significant, moderate, and positive correlation between the B-IPQ and MFIS-BR total score, as well as physical and psychosocial domains, ranging from 0.578 to 0.672, which was substantially larger than the correlation for the cognitive domain (r = 0.360; *p* = 0.051). Thus, higher MFIS-BR scores, indicating greater levels of overall fatigue, particularly physical and psychosocial fatigue, were associated with higher B-IPQ scores, indicating greater threat perception of the disease ( Figure 3 ).

Significant correlations were also observed between illness perception (B-IPQ) and both domains of the quality-of-life assessment scale (MSQOL-54). As shown in Figure 3 , the correlations between B-IPQ and MSQOL-54's physical health (r = −0.776; *p* < 0.001) and mental health (r = −0.704; *p* < 0.001) domain were strong and negative. This indicates that higher MSQOL-54 scores, representing better quality of life in physical and mental health, were associated with lower B-IPQ scores, meaning lower threat perception of the disease.

In the studied population, neither disease duration ( *p* = 0.419) nor EDSS score ( *p* = 0.330) was significantly associated with the perception of a relevant threat. However, in the model analyzing B-IPQ scores, EDSS was significant ( *p* = 0.034), as shown in Table 2 . Thus, after adjusting for disease duration, a 1-point increase in the EDSS score resulted in an average increase of 2.30 points in the B-IPQ score, indicating that higher levels of disability are associated with a greater threat perception of the disease.

**Table 2:** Effects of disease duration and EDSS on B-IPQ score and illness perception status

Total (n = 30)	B-IPQ scores		Relevant threat perception	
	Adjusted coefficient (95%CI)	*p* -value	Adjusted OR (95%CI)	*p* -value
**Disease duration (years)**	−0.12 (−0.55–0.32)	0.592	0.96 (0.86–1.06)	0.419
**EDSS**	2.30 (0.19–4.40)	**0.034**	1.28 (0.78–2.11)	0.330

Abbreviations: B-IPQ, Brief Illness Perception Questionnaire; EDSS, Expanded Disability Status Scale; OR, odds ratio.

Notes: The effects of disease duration and EDSS on B-IPQ continuous score were evaluated using multivariate linear regression (R ^2^ = 15.6%). The effects of disease duration and EDSS on illness perception status, measured by outcome of relevant threat perception (B-IPQ > 33) were evaluated using multivariate logistic regression (pseudo-R ^2^ = 0.023). A 5% significance level ( *p* < 0.05) was adopted.

## DISCUSSION

Our study did not demonstrate a significant correlation between cognitive performance and illness perception, indicating that cognitive impairment may not influence how patients perceive their disease. Interestingly, our population did not exhibit cognitive impairment based on the cutoff score established by the ABN for the MoCA-BR test. 11

Curatoli et al. 18 reached a similar conclusion. They evaluated factors affecting perceived disability using the World Health Organization Disability Assessment Schedule 2.0 (WHODAS 2.0), a questionnaire developed to comprehensively assess disability, incorporating both psychosocial and environmental factors. In their study, cognitive performance was also not significantly associated with perceived disability. Similar to our research, their population had a low prevalence of cognitive impairment. 18

Unlike our study, Curatoli et al. 18 used the Italian version of the Brief Repeatable Battery of Neuropsychological Tests (BRB-NT), an internationally validated battery of cognitive tests specifically assessing functions commonly affected in MS. It consists in five tests: Selective Reminding Test (SRT), Symbol Digit Modalities Test (SDMT), Paced Auditory Serial Addition Task (PASAT), Controlled Oral Word Associtation Test (COWAT) and 10/36 Spatial Recall Test (10/36-SRT). However, Curatoli et al. chose to use only SRT, SDMT and PASAT. 18

The present study used the MoCA-BR scale because it is widely used as a screening tool for detecting mild cognitive impairment. Furthermore, it has been validated as a sensitive instrument for identifying MS-related cognitive impairment, assessing cognitive domains commonly affected in this condition. 19 Moreover, the MoCA-BR scale offers greater objectivity and ease of administration, especially given that participants were assessed using multiple scales simultaneously, which may have diminished their engagement.

It is known that patients with cognitive impairment due to other neurodegenerative conditions may exhibit anosognosia, defined as an inability to recognize one's deficits. 20 Anosognosia can affect an individual's perception of their illness, as the belief of not having any deficits leads to a distorted understanding of the condition, preventing them from recognizing the potential threat of their disease.

Some researchers have investigated whether this phenomenon also occurs in MS. Recent studies suggest a nonlinear relationship between patients' subjective perceptions and their performance on neuropsychological tests, indicating that patients with mild deficits tend to overestimate their impairments. In contrast, those with more severe cognitive impairment tend to underestimate them. 6 Thus, anosognosia could occur in individuals with greater cognitive impairment, leading to a false perception of absent threat. However, in our study, this was not observed, given that the average cognitive performance, as assessed by the MoCA score, fell within the normal range.

On the other hand, our study demonstrated that fatigue directly impacts illness perception, with higher levels of fatigue correlating with a greater perceived threat of the disease. Our results are consistent with those reported by Bassi et al.. 8 They analyzed the correlation between each subitem of the Illness Perception Questionnaire (IPQ-R) and fatigue, finding that the identity, timeline, and consequences subitems were associated with higher levels of fatigue. In comparison, the treatment control subitem was associated with lower levels of fatigue. 8

When analyzing the correlation between each fatigue domain and illness perception, we found a significant correlation in all domains except the cognitive domain. This result is reassuring and supports our initial findings, suggesting that cognition does not influence illness perception, nor does cognitive fatigue.

We observed that quality of life directly affects illness perception in both the physical and mental health domains, with higher scores being associated with a lower perceived threat from the disease. Our results were consistent with previous studies, which have shown that quality of life and illness perception are interrelated and influence each other. 8 21 22 23 Finally, we demonstrated that the degree of disability influences illness perception; the higher the neurological disability, the greater the perceived threat. This finding is similar to that of Curatoli et al., as previously mentioned. 18

The main limitation of our study was the inability to evaluate patients with greater cognitive impairment, as dementia was an exclusion criterion. We applied multiple scales simultaneously, requiring a higher level of cognitive understanding. Therefore, participants with established cognitive deficits were excluded. Furthermore, performing multiple tests can reduce patient engagement, leading to fatigue bias.

Another point was the utilization of the MoCA test. Although a validated instrument, it may not have fully captured the nuances of cognitive impairment in MS. The lack of information about mood and anxiety also limits the complete understanding of illness perception in our study, since these aspects can influence patients' resilience in coping with the disease. We acknowledge that, due to the small sample size and the study design, it is not possible to draw definitive or causal conclusions; however, for a pilot study, our findings are consistent.

In conclusion, illness perception in MS is influenced by fatigue, quality of life, and disability levels, but it does not appear to be affected by cognition. Understanding how pwMS perceive their disease and identifying the factors influencing this perception is crucial for optimizing coping strategies and treatment adherence.
